# A Single Faecal Microbiota Transplantation Altered the Microbiota of Weaned Pigs

**DOI:** 10.3390/life10090203

**Published:** 2020-09-15

**Authors:** Tanya L. Nowland, Valeria A. Torok, Wai Y. Low, Kate J. Plush, Mary D. Barton, Roy N. Kirkwood

**Affiliations:** 1 School of Animal and Veterinary Sciences, The University of Adelaide, Roseworthy 5371, Australia; valeria.torok@sa.gov.au (V.A.T.); roy.kirkwood@adelaide.edu.au (R.N.K.); 2 Food Sciences, South Australian Research and Development Institute, Waite Campus, Urrbrae 5064, Australia; 3 The Davies Research Centre, School of Animal and Veterinary Sciences, The University of Adelaide, Roseworthy 5371, Australia; wai.low@adelaide.edu.au; 4 SunPork Group, Murarrie 4172, Australia; kate.plush@sunporkfarms.com.au; 5 School of Pharmacy and Medical Sciences, University of South Australia, Adelaide 5000, Australia; mary.barton@unisa.edu.au

**Keywords:** pigs, weaning, enteric dysbiosis, microbiota transplantation, enteric microbiota

## Abstract

Weaning is a stressful time for piglets, often leading to weight loss and is associated with increased morbidity and mortality. A leading cause for these post-weaning problems is enteric dysbiosis and methods to improve piglet health at this crucial developmental stage are needed. This study aimed to determine whether an enteric dysbiosis caused by weaning could be corrected via a faecal microbiota transplantation (FMT) from healthy piglets from a previous wean. Two or four focal piglets per litter were assigned to one of two treatments; FMT two days post weaning (*n* = 21; FMT) or a control which received saline two days post weaning (*n* = 21; CON). FMT consisted of homogenised donor faeces administered orally at 3 mL/kg. Weaning occurred at 18 days of age and weights and faecal samples were collected on days 18, 20, 24 and 35. 16S rRNA amplicon analysis was used to assess the faecal microbiota of piglets. FMT increased Shannon’s diversity post weaning (*p* < 0.001) and reduced the scratch score observed at 24 days of age (*p* < 0.001). The bacterial populations significantly differed in composition at each taxonomic level. In FMT pigs, significant increases in potentially pathogenic *Escherichia coli* were observed. However, increases in beneficial bacteria *Lactobacillus mucosae* and genera *Fibrobacteres* and *Bacteroidetes* were also observed in FMT treated animals. To our knowledge, this is the first study to observe a significant effect on piglet faecal microbiota following a single FMT administered post weaning. Therefore, FMT post weaning can potentially alleviate enteric dysbiosis.

## 1. Introduction

Weaning is one of the most stressful times in a piglet’s life. It involves separation from the sow, a change in diet from primarily milk to solid feed and the mixing of litters [1]. As a result of this stress, piglets have reduced daily feed intake following weaning which results in villus atrophy, increased intestinal permeability and may result in post-weaning diarrhoea (PWD) [2]. Much research has investigated ways for making the weaning transition easier for piglets, including provision of creep feed during lactation [3] and the inclusion of ZnO or in-feed antibiotics in post weaning diets as a preventative for PWD [4,5]. Research has demonstrated some benefit from creep feeding; however, with weaning ages as young at 18 to 21 days, the impact of feeding creep is minimal [3]. While ZnO provides benefits to piglets by reducing PWD and increasing growth performance, it is an environmental pollutant and as such its use is restricted in some countries [6]. Both antibiotics and high levels of ZnO are associated with antimicrobial resistance [7] and this necessitates the development of alternatives.

Microbiota are communities of microorganisms colonising all body surfaces. These microorganisms include bacteria, fungi, archaea and viruses [8,9]. The microbiota within the gastrointestinal tract has been demonstrated to be involved in nutrient metabolism and immune system development and function, and disruptions of the microbiota cause long-term health problems [10]. Some of these problems include necrotising enterocolitis, a reduced immune response following infection and an associated higher susceptibility to disease, reduced growth and diarrhoea in pigs [11,12,13,14]. As such, the development or maintenance of a healthy gut microbiota is important for health and survival.

One method that has been successful for the treatment of enteric diseases such as *Clostridium difficile* infections in humans is faecal microbiota transplantation (FMT) [15]. FMT was first described by Ge Hong in fourth century China for treating food poisoning and severe diarrhoea [16]. FMT involves transferring faeces from a healthy donor to a sick recipient with the aim of re-establishing a healthy microbiota through the competitive exclusion of pathogenic bacteria. The aim of the present study was to determine whether a dysbiosis caused by weaning could be corrected via FMT from healthy piglets from a previous weaning. We hypothesised that the microbiota of the donor pigs would be in a state optimal for the post-weaning changes of diet and environment, therefore providing a benefit to the naïve, newly weaned piglet. As a result, the weaning stress associated with enteric dysbiosis would be corrected by administration of FMT post weaning.

## 2. Materials and Methods

All procedures were conducted at the University of Adelaide Roseworthy piggery with the approval of the University of Adelaide’s Animal Ethics Committee (AEC number: S-2017-063).

### 2.1. Experimental Design and Sample Collection

A total of 19 Large White x Landrace sows (parities 2 to 4; mean ± standard deviation: 2.8 ± 0.8) were used in the study. All sows were group housed during gestation and received no antibiotics. Sows were moved into the farrowing house at day 110 of gestation where they received a commercial lactation diet (14.2 MJ DE/kg) twice daily and had free access to water. Prior to farrowing, sows were fed 2.5 kg/d, which was gradually increased to 7 to 8 kg/d by day 7 after farrowing. Two days before their due date, sows were induced to farrow using split-dose vulva injections of cloprostenol (125 µg at 7 a.m. and 2 p.m.). At weaning (18 days of age), 2 or 4 male piglets per sow were selected at random to be focal pigs and were assigned equally to one of two treatments:

- FMT: Faecal transplant administered two days post weaning (*n* = 21; FMT)
- Control: Saline administered two days post weaning (*n* = 21; CON)

**Figure 1:**
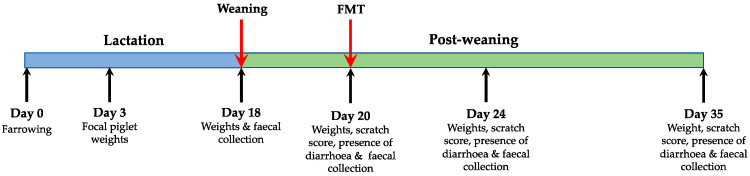
Schematic of the experimental timeline. Piglets were divided into two groups at weaning, control animals (CON; *n* = 21) or faecal microbiota transplantation (FMT; *n* = 21), the FMT procedure is described in the materials and methods section. On day 3, 18, 20, 24 and 35, all piglets were weighed. Faecal samples, scratch scores and diarrhoea incidence were collected from day 18 onwards as stated in the figure above.

To ensure that the effect of sow was not confounding, the focal piglets per litter were assigned equally to treatments. Sows farrowed over two days and piglets were cross fostered to achieve 10 or 11 piglets per litter 24 h post-partum. Weaning occurred on day 18. Focal piglets were weaned into two pens with slatted flooring according to their treatment. Both pens were in the same room and had a walkway separating them to prevent faecal transfer between pens. Heat lamps were set over one corner of the pens and feeders were placed in the other. Piglets had *ad libitum* access to antibiotic-free weaner feed (14.5 MJ DE/kg) and water throughout the experiment. All focal piglets were individually weighed at 3, 18, 20, 24 and 35 days of age. Faecal samples were collected from each focal piglet at weaning (18 days), prior to FMT (20 days of age) and 4 and 15 days post FMT (age 24 and 35 days). Faeces were placed on ice immediately, transported to the laboratory within 4 h and stored at −80 °C. For FMT, donor faeces were collected from eight clinically healthy male piglets from a previous weaning (7 weeks old) and blended 1:2 with saline. Donor piglets were housed in a slatted floored pen with *ad libitum* access to antibiotic-free feed and had no previous contact with antibiotics or antibiotic-treated animals. All faecal blends were collected within an hour of FMT and were at room temperature before being delivered by oral gavage at 3 mL/kg [16]. Piglets were fasted for 3 h before FMT to minimise gastric acidity. All piglets were monitored for any adverse reactions, such as vomiting, but none were noted. Scratch scores as an indicator of fighting and the presence of diarrhoea were recorded each time the piglets were weighed post weaning. Diarrhoea was defined as the presence of liquid faeces as described previously [17] and scratch scores were recorded based on the system used by Seyfang et al. [18]. A schematic of the experimental timeline can be seen in Figure 1.

### 2.2. DNA Extraction and 16S rRNA Amplicon Sequencing

Total nucleic acid was extracted from freeze dried piglet faecal samples using a proprietary method (South Australian Research and Development Institute, Adelaide, Australia) [19,20,21]. The V3–V4 region of the 16S rRNA gene was sequenced using the illumina MiSeq platform using 300 bp paired end reads (forward primer: CCTAYGGGRBGCASCAG and reverse primer: GGACTACNNGGGTATCTAAT), following a standard protocol by the Australian Genome Research Facility for next generation sequencing (Melbourne, Australia). The obtained reads are available under the accession number PRJNA634575 of the Sequence Read Archive of the NCBI. For bioinformatic analysis of raw sequence data performed by AGRF, the paired-end sequences were merged by aligning the forward and reverse reads using PEAR v0.9.5 [22] and the primers were identified and trimmed. All trimmed sequences were processed using Quantitative Insights into Microbial Ecology (QIIME 1.8) [23], USEARCH (version 8.0.1623) [24,25], and UPARSE software [26]. Sequences were quality filtered and full-length duplicate sequences were removed and sorted by abundance. Singletons or unique reads in the dataset were discarded. Additionally, chimeric sequences were clustered and removed using the “rdp_gold” database as the reference. Sequences were grouped into operational taxonomic units (OTUs) based on 97% sequence similarity. Using QIIME, taxonomy was assigned using the Silva database v132 [27].

### 2.3. Statistical Methods

All data were tested for the normality of residuals and outliers before analysis, no outliers were found or removed, and all weight and average daily gain data were normally distributed. Outliers were defined as data points greater than two standard deviations from the mean and were deemed as aberrant data. All weight, average daily gain, diarrhoea incidence and scratch score data were analysed using SPSS, v26 (IBM, Armonk, NY, USA). A linear mixed model with repeated measures design was used to assess the effect of treatment on piglet weight and average daily gain. The fixed effects included in the model were treatment, age and treatment*age, with age specified as the repeated measure. Scratch score data were analysed using a generalised linear model, repeated measures design with a Poisson distribution, and diarrhoea incidence data were analysed using a generalised linear model, repeated measures design with a binary logistic regression using the same model. Data were expressed as estimated marginal means ± standard error of the mean, and a *p*-value threshold of 0.05.

Multivariate statistical techniques (PRIMER6, PRIMER-E Ltd., Ivybridge, UK) were used to analyse the faecal 16S rRNA bacterial taxonomic data. Similarities between faecal bacterial communities of piglets from the 16S rRNA data metrics were analysed using Bray–Curtis measures of similarity [28], following standardisation and fourth-root transformation. Analysis of similarity (ANOSIM) [29] was used to test if there were significant treatment and age differences between faecal bacterial communities. In order to determine which individual bacterial taxa contributed most to the overall dissimilarity among treatment and age groups, similarity percentages (SIMPER) [29] analyses were done and the overall average dissimilarity between piglet faecal bacterial communities were calculated. The contributions (%) of significant OTUs (average dissimilarity/standard deviation > 1) to the top 60% of the average dissimilarities were calculated. Non-metric multidimensional scaling (nMDS) [30,31] was done to graphically illustrate relationships between ages.

Alpha diversity metrics; Shannon diversity (H’) index, Pielou’s evenness (J’) and number of taxa (S) were calculated using DIVERSE (PRIMER6 PRIMER-E Ltd., Ivybridge, UK). Normality was tested within RStudio software (Version 1.1.456, Boston, MA, USA) using the Shapiro–Wilk test. Those alpha diversity metrics that were found to be normally distributed were analysed using an analysis of variance (ANOVA) and those not normally distributed were analysed using the Kruskal–Wallis test, with corrections for multiple tests using false discovery rate (FDR) and a *p*-value threshold of 0.05.

## 3. Results

### 3.1. Production Characteristics

No significant differences in piglet weights were evident between treatments at 24 (CON: 6.8 ± 0.2 kg, FMT: 6.5 ± 0.2 kg; *p* = 0.391) and 35 (CON: 9.0 ± 0.3 kg, FMT 8.9 ± 0.3 kg; *p* = 0.776) days of age. Average daily gain was higher for control animals from 20 to 24 days of age (CON: 0.07 ± 0.01 kg, FMT: 0.02 ± 0.01 kg; *p* = 0.003), but no significant difference between treatments was observed for average daily gain between 24 and 35 days of age (CON: 0.20 ± 0.02 kg, FMT: 0.21 ± 0.02 kg; *p* = 0.664). A post-weaning growth check was observed for piglets regardless of treatment; average daily again from 3 days old to weaning (18 days) was 0.30 ± 0.01 kg, from weaning to 20 days was −0.12 ± 0.01 kg, 20 to 24 days old was 0.04 ± 0.01 kg and from 24 to 35 days of age was 0.21 ± 0.01 kg. No significant difference for the occurrence of diarrhoea was observed at 24 days or 35 days of age (25% CI (14, 40) and 0.9% CI (0.01, 88), respectively; *p* = 0.385). Those animals treated with a faecal transplant had significantly lower scratch scores at 24 days of age when compared to control animals (CON: 0.86 ± 0.19, FMT: 0.05 ± 0.04; *p* < 0.001).

### 3.2. Age Related Effects

**Figure 2:**
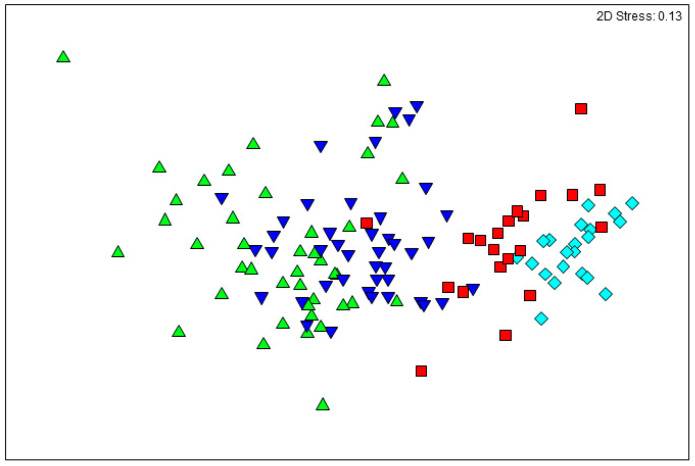
nMDS ordination of faecal bacterial genera from piglets at 18 (triangle), 20 (inverted triangle), 24 (square) and 35 (diamond) days of age. nMDS ordinations attempt to place all samples in an arbitrary two-dimensional space such that their relative distances apart match the corresponding pairwise similarities. Hence, the closer the two samples are in the ordination, the more similar their overall bacterial communities. “Stress” values (Kruskal’s formula 1) reflect the difficulty involved in compressing the sample relationship into the two-dimensional ordination.

**Figure 3:**
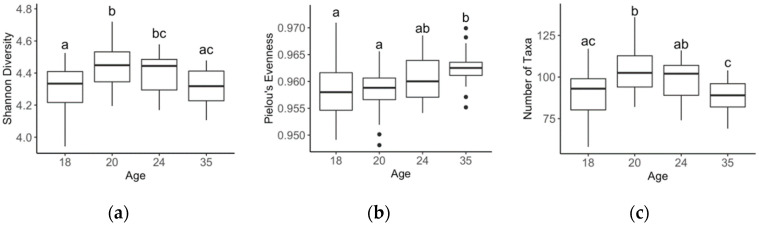
Comparison of Shannon diversity (**a**), Pielou’s evenness (**b**), and number of taxa (**c**) between piglets at age 18, 20, 24 and 35 days at the genus level. Means with different superscripts (a, b, c) are significantly different (*p* < 0.05).

On average, 50,578 16S rRNA sequenced reads were retained after quality control per faecal sample. Faecal bacterial genera significantly differed as piglets aged, with all age-wise comparisons being different (Global R = 0.538, *p* < 0.001; Figure 2). Alpha diversity metrics such as Shannon’s diversity, Pielou’s evenness and number of taxa, differed between ages (*p* < 0.001; Figure 3). Shannon’s diversity and number of taxa increased from 18 to 20 days of age (*p* < 0.001), thereafter decreasing to levels similar to 18-day-old animals by 35 days of age. Pielou’s evenness increased significantly as piglets aged (*p* < 0.001; Figure 3).

**Figure 4:**
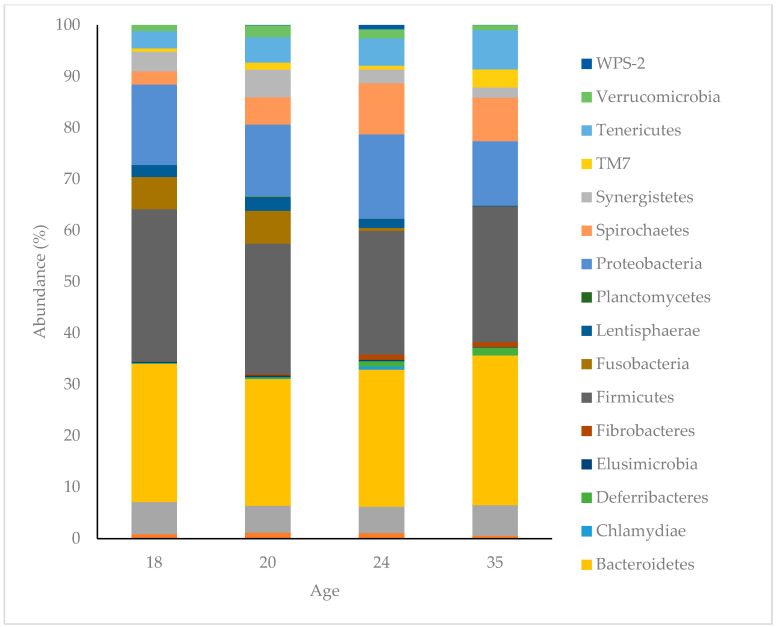
Abundance (%) of bacterial phyla present in the faeces of pigs at 18, 20, 24 and 35 days of age. The bacterial phlya within the legend are arranged in the same order as they appear in the bar chart.

The dominant phyla in the faecal microbiota of piglets at 18 days of age were Bacteroidetes (26.69%), Firmicutes (29.72%), Proteobacteria (15.67%), Fusobacteria (6.30), Actinobacteria (6.22%) Synergistetes (3.78%), Tenericutes (3.34%), and Spirochaetes (2.61%). These made up 94.55% of all bacteria found in piglet faeces at 18 days of age, with 10 bacterial phyla comprising the remaining 5.45% (Figure 4). The proportions of these phyla changed with age, collectively representing 91.51%, 90.95%, 92.24% at 20, 24 and 35 days of age, respectively. The three most dominant phyla represented as piglets aged (Bacteroidetes, Firmicutes and Proteobacteria) remained the same; however, Fusobacteria, which was the fourth most abundant phylum at 18 and 20 days of age, declined and was not represented at 35 days of age. The average dissimilarity in bacterial phyla between age groups ranged from 18 to 24%. The main phyla driving significant change between 18 and 35 days of age were increases in Spirochaetes, Tenericutes, TM7, Bacteroidetes, Deferribacteres and Fibrobacteres, and decreases in Fusobacteria, Proteobacteria, Synergistetes, Lentisphaerae and Firmicutes at 35 days of age.

At the genus level, the average dissimilarity in faecal microbiota between 18- and 35-day-old piglets was 44.21%. Of the genera significantly contributing to the top 60% of dissimilarity, *Bacteroides, Butyricimonas, Escherichia, Parabacteroides, Fusobacterium, Lactobacillus, Bilophila, Oscillospira, Enterococcus, Clostridium, Ruminococcus, Veillonella, Streptococcus, Rothia* and *Collinsella* were more abundant at 18 days and *Roseburia, Prevotella, Lachnospira, Succinivibrio, Lachnobacterium, Treponema, Anaerovibrio, Sarcina, Bulleidia, Coprococcus, Butyrivibrio, Mitsuokella, Megasphaera, Faecalibacterium, Campylobacter, Acidaminococcus, Catenibacterium, Turicibacter* and *Dialister* were more abundant at 35 days. Of the taxa which could be classified to the species level, *Bacteroides fragilis, Escherichia coli, Parabacteroides distansonis, Lactobacillus delbrueckii, Clostridium perfringens, Streptococcus luteciae, Clostridium hathewayi*, and *Bacteroides uniformis* were more abundant in the 18 day old piglets and *Prevotella copri, Roseburia faecis, Faecalibacterium prausnitzii, Prevotella stercorea, Ruminococcus bromii,* and *Ruminococcus flavefaciens* were more abundant in 35 day old piglets, contributing to the top 50% of dissimilarity between these groups.

### 3.3. Donor Sample Composition

The main phyla present within the pooled homogenate of eight donor pigs were Actinobacteria, Bacteroidetes, Fibrobacteres, Firmicutes, Proteobacteria, Spirochaetes, TM7 and Tenericutes. Of the taxa that could be identified to genus level, the top 6 bacteria present were *Prevotella* (60.16%), *Roseburia* (6.36%), *Oscillospira* (2.63%), *Faecalibacterium* (1.7%), *Lachnospira* (1.54%) and *Dialister* (1.06%), while 53 other genera comprised the remaining bacteria present and all individually represented less than 1% of the total bacteria present. Of the taxa that could be identified to species level, the donor sample homogenate contained *Prevotella ruminicola, Streptococcus luteciae, Defluviitalea saccharophila, Bacteroides plebeius, Helicobacter equorum, Clostridium hathewayi, Asteroleplasma anaerobium, Coprococcus eutactus, Ruminococcus callidus, Oxalobacter formigenes, Clostridium piliforme, Eubacterium biforme, Mitsuokella multacida, Lactobacillus reuteri, Butyricicoccus pullicaecorum, Ruminococcus gnavus, Faecalibacterium prausnitzii, Roseburia faecis, Collinsella aerofaciens, Escherichia coli, Eubacterium cylindroides, Lactobacillus mucosae, Ruminococcus flavefaciens, Ruminococcus bromii, Desulfovibrio D168, Prevotella stercorea* and *Prevotella copri.*

### 3.4. Treatment by Age Effects

**Figure 5:**
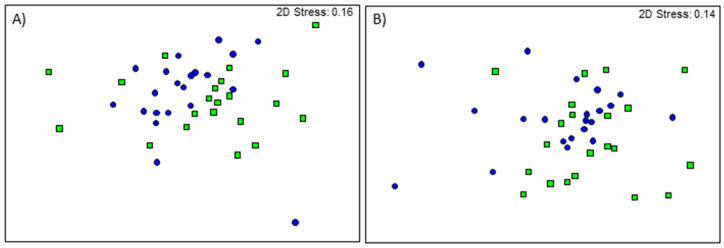
nMDS ordination of faecal bacterial genera from control (square) and FMT (circle) piglets at (**A**) 24 days of age and (**B**) 35 days of age.

**Figure 6:**
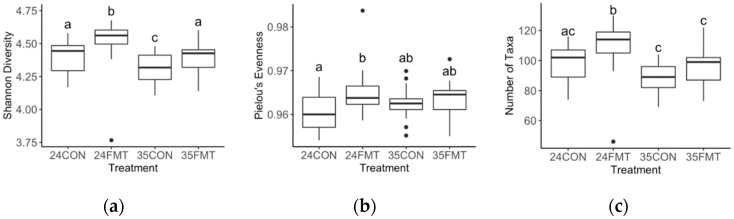
Comparison of Shannon diversity (**a**), Pielou’s evenness (**b**), and number of taxa (**c**) between piglets in treatments CON and FMT at 24 and 35 days of age at the genus level. Means with different superscripts (a, b, c) are significantly different (*p* < 0.05).

Faecal bacterial genera differed between treatments at 24 (Global R = 0.168, *p* = 0.002; Figure 5) and 35 days of age (Global R = 0.110, *p* = 0.001; Figure 5). For alpha-diversity metrics, Shannon’s diversity was also different between treatments in both age groups (*p* < 0.001; Figure 6), while Pielou’s evenness and number of taxa only differed with treatment in the 24-day-old piglets (*p* = 0.040 and *p* < 0.001, respectively; Figure 6).

The main phyla driving the change between treatments at 24 days of age were increases in Spirochaetes, Deferrbacteres, Synergistetes, TM7, Elusimicrobia, WPS-2, Fibrobacteres, Tenericutes, Firmicutes, Verrucomicrobia and Lentisphaerae, and decreases in Proteobacteria, Bacteroidetes and Chlamydiae for those animals treated with FMT. The main phyla driving the difference between treatments at 35 days of age were an increase in Proteobacteria, Deferribacteres, Fibrobacteres, Elusimicrobia, Actinobacteria and Bacteroidetes and decreases in Spirochaetes, TM7, Synergistetes, Terenicutes, Verrucomicrobia and Firmicutes for those animals treated with FMT.

The average dissimilarity in bacterial genera between treatments in piglets at 24 days of age was 27% (Table 1). Of those taxa which could be classified to genus level and significantly contributed to the top 60% of dissimilarity, *Acidaminococcus, Dorea, YRC22, Butyrivibrio, Pyramidobacter, Mucispirillum, Streptococcus, Actinobacillus, Anaerovibrio, Butyricimonas, Oscillospira, Lachnobacterium, SMB53, Fibrobacter, Lachnospira* and *rc4-4* were more abundant in animals treated with the faecal transplant and *Succinivibrio, Prevotella, Mitsuokella, Lactobacillus, Faecalibacterium, Megasphaera, Catenibacterium, Collinsella* and *Roseburia* were more abundant in control animals of the same age. The average dissimilarity in bacterial genera between treatments in piglets aged 35 days was 22% (Table 2). Of those taxa which could be classified to genus level and significantly contributed to the top 60% of dissimilarity, *Dialister, Shuttleworthia, Acidaminococcus, Mitsuokella, Campylobacter, Megasphaera, Catenibacterium, Bulleidia, Streptococcus, Mucispirillum, Actinobacillus, Anaerostipes, Faecalibacterium, Escherichia, Ruminococcus, Fibrobacter, Collinsella, Oscillospira* and *L7A_E11* were more abundant in animals treated with the faecal transplant and *Succinivibrio, Treponema, Lachnobacterium, Sarcina, Prevotella, Roseburia, Phascolarctobacterium, Parabacteroides, YRC22, rc4-4, SMB53, Lachnospira, Dorea, p-75-a5, CF231, Oxalobacter* and *Coprococcus* were more abundant in control animals.

Of those taxa which could be classified to the species level at 24 days, *Escherichia coli, Desulfovibrio D168, Ruminococcus flavefaciens, Mucispirillum schaedleri, Eubacterium cylindroides, Lactobacillus mucosae* and *Streptococcus luteciae* were more abundant in animals treated with the faecal transplant and *Prevotella copri, Prevotella stercorea, Faecalibacterium prausnitzii, Ruminococcus bromii, Mitsuokella multacida, Sharpea azabuensis, Collinsella aerofaciens, Roseburia faecis, Ruminococcus callidus* and *Helicobacter equorum* were more abundant in control animals, contributing significantly to the top 60% of dissimilarity between these treatment groups. Of those taxa which could be classified to the species level in 35 day old pigs, *Prevotella copri, Desulfovibrio D168, Ruminococcus bromii, Lactobacillus mucosae, Mucispirillum schaedleri, Eubacterium cylindroides, Prevotella stercorea, Faecalibacterium prausnitzii, Escherichia coli, Collinsella aerofaciens* and *Clostridium hathwayi* were more abundant in animals that had been treated with a faecal microbiota transplant and *Mitsuokella multacida, Roseburia faecis, Ruminococcus flavefaciens, Lactobacillus reureri, Ruminococcus callidus, Oxalobacter formigenes* and *Coprococcus eutactus* were more abundant in control animals, contributing to the top 60% of dissimilarity between these treatment groups.

## 4. Discussion

Our data support the hypothesis that weaning stress would cause enteric dysbiosis that is corrected through FMT post weaning and, to our knowledge, this is the first study investigating FMT in pigs that has demonstrated an effect after a single FMT post weaning.

It is accepted that an increase in microbial diversity is beneficial for the gut health of an individual (for reviews, see: [10,32,33]). The greater the diversity of bacteria present, the less chance pathogenic bacteria have to colonise and cause disease. It is not the presence of pathogenic bacteria that causes disease, but rather whether they proliferate to an extent that overwhelms the commensal microbial population [32]. In the present study, treating piglets with FMT post weaning resulted in a significant increase in faecal microbiota diversity at both 24 and 35 days of age. This increase in diversity likely indicates that the piglets within the FMT treatment may have an improved ability to cope with and adapt to the challenges associated with weaning. Indeed, this was corroborated by the observation of an increase in bacterial diversity and improvements in goblet cell mucin stores, and a reduced necrotising enterocolitis incidence in those pigs treated with FMT during the first few days of life [34]. Furthermore, Geng et al., [35] observed improvements in bacterial diversity and a reduced susceptibility to epithelial injury in those piglets treated with FMT for the first 14 days of life. Both studies demonstrated the positive implications increased microbial diversity can have on intestinal barrier function.

The gastrointestinal tract (GIT) microbiota undergoes dysbiosis post weaning, which is a leading cause of increased GIT permeability and PWD, both of which are associated with an increase in mortality and a reduction in feed intake and growth in pigs, known as a post-weaning growth check [1]. In common with the prior literature, the pigs within this study also showed a post-weaning growth check irrespective of treatment. Although the treatment did not improve body weight in the present study, it provided no hinderance either. It is possible that the increase in diversity caused by the FMT provided benefits to the piglets that were not observable with the current animal numbers. Average daily gain was reduced in FMT-treated animals at 24 days of age (four days post FMT); however, this was somewhat expected as the piglets receiving the FMT would have had to undergo some adjustment to the rapidly changing GIT microbiota. Incidence of PWD did not differ between treatments at this time either, while scratch score was reduced in FMT-treated pigs, indicating no negative effect on the GIT and a reduction in piglet fighting. Fighting at weaning is common and can add to the stress associated with the weaning event; therefore, the fact that the treatment reduced this may provide additional benefits. Studies have indicated that behaviour is influenced via the gastrointestinal microbiota, so its alteration via FMT may partially explain the outcome observed [36]. By 35 days of age, there was no significant difference in ADG between treatments. As no differences in weight were observed at any time-point, it indicates no long-lasting negative effect of FMT on piglet growth. The present study differs from previous studies in that FMT was conducted post weaning and piglets were only dosed once. Earlier works [17,37] observed positive implications for piglet health by demonstrating both an improvement in average daily gain and a reduction in diarrhoea incidence during lactation when multiple FMTs were administered to piglets in early life. It is likely that no effect on the incidence of diarrhoea was observed in the present study because the animals were weaned into pens of 21 piglets rather than the large numbers often seen in commercial production and as such, the incidence of diarrhoea was relatively low in our study.

*Escherichia coli* is the primary infectious agent of PWD in piglets [1] and, although the piglets treated with FMT in the present study had a higher abundance of *E. coli* in their faeces at 24 and 35 days of age, no difference in diarrhoea incidence was observed. Not all strains of *E. coli* are pathogenic, with some having probiotic qualities [38,39], and as 16S rRNA amplicon analysis does not distinguish between strains, it was not possible to determine whether the *E. coli* observed in the present study were beneficial or potentially pathogenic. The presence of *E. coli* in this instance may have provided benefits to the microbiota stability, as a reduction in serotype diversity may allow for a pathogenic monoculture to develop rather than a beneficial one. Additionally, as previously mentioned, infection is not only due to the presence of a pathogenic bacteria but occurs when numbers proliferate to an extent that overwhelms the commensal microbial population. Therefore, it is likely that if the *E. coli* present included a pathogenic strain, the potential pathogenic nature of the bacteria may have been negated by the presence of other bacteria. For example, *Lactobacillus mucosae* was one bacterium that was more abundant in FMT-treated animals at 24 and 35 days of age. *Lactobacillus mucosae* is known for its ability to competitively attach to the epithelium of the intestine, produce antimicrobials and inhibit pathogenic bacteria and, therefore, could have provided additional benefit in preventing the potential pathogenic properties of *E. coli* [40].

Those animals receiving FMT also had an increase in beneficial Fibrobacteres and Bacteroidetes at 35 days of age. Fibrobacteres are known as adept fibre degraders which could provide great benefit to the weaned piglet as weaning introduces a large dietary change from primarily milk to solid food [41]. Therefore, it is not surprising that FMT-treated animals had a higher abundance of Fibrobacteres as it was in the donor faeces given and would have provided these animals with a microbiota more developed for the digestion of solid feed. Additionally, the increased presence of Bacteriodetes in FMT-treated piglets at 35 days of age may have also provided benefits to piglets; a higher relative abundance of Bacteroidetes in healthy pigs after weaning compared with those that developed diarrhoea has been observed [42]. Additionally, control animals had a higher relative abundance of *Prevotella* at 24 days of age, which may explain the improvements in average daily gain observed at this age. Previous studies have documented *Prevotella* to have a positive correlation with body weight [43]. However, by 35 days of age, no difference in average daily gain was observed between treatments, while *Prevotella* remained more abundant in control animals. Interestingly, the donor faeces contained large amounts of *Prevotella,* therefore the opposite would have been expected. *Treponema,* a potentially pathogenic bacteria which is associated with swine dysentery [44] was present in higher amounts in control piglets at 35 days of age, which may have reduced the benefits of *Prevotella*. These results demonstrate the importance of community composition rather than the presence or absence of any particular bacteria.

Recently, studies investigating the use of FMT in pigs for the improvement of health and production outcomes have increased in number. However, current techniques can be quite stressful for the pig as they involve administration of multiple faecal doses over a number of days and use interventions to reduce gastric acid in order to improve post-gastric bacterial survival. The results from this study are the first to indicate that FMT is successful if done post weaning and that minimal intervention is needed in order to influence the microbiota of piglets. Only two previous studies administered a single dose, as opposed to the multi-dose approach usually implemented, and these studies were conducted during lactation. McCormack et al., [45] administered a dose at birth and observed a negative impact on growth and an altered faecal microbiota, while our research group administered a single dose at 7 days of age and saw no effect on the faecal microbiota [46]. This is likely because the GIT of a newly born piglet is relatively immature and hence would be susceptible to colonisation, while a 7-day-old piglet would have bacteria already colonised which would compete directly with the FMT. Our data indicate that weaning caused a large enough disruption of the microbiota to enable the colonisation of new bacteria, as is likely the cause of PWD. While it makes for an optimal time for pathogenic bacterial colonisation, it also offers an appropriate time to implement a treatment targeted at influencing positive microbiota development, such as FMT.

When assessing the effect of weaning on the microbiota of piglets, it is evident that diversity does not increase linearly with age. In the present study, Shannon’s diversity and the number of taxa increased from weaning to 2 days post weaning and, thereafter, by 35 days decreasing to levels similar to those observed on day 18, while evenness increased steadily from 18 to 35 days of age. The reduction in bacterial diversity observed post weaning is likely the result of dietary and environmental changes induced by weaning. Similarly, an earlier review documented weaning’s negative impact on the GIT microbiota and its impact on alterations to the resident bacteria [47]. Therefore, the change in microbiota we observed may be due to GIT stabilisation during the post-weaning period. Hence, it is probable that the increase in diversity observed at 20 days of age are when the bacteria needed for milk and solid feed digestion coexist. Thereafter, the microbiota required for the digestion of solid feed remain while those bacteria required for milk digestion decline, resulting in a drop in diversity. This GIT stabilisation can also be observed when looking at the variability between piglets at each age stage. It is evident that as age increases, the variability between piglets decreases. This is to be expected as the influence of the sow is removed at weaning and all piglets are housed in the same environment on the same feed.

## 5. Conclusions

Our findings document that FMT significantly affected the piglet’s microbiota post weaning and reduced the scratch scores observed at 24 days of age. As such, our data suggest that administering FMT after weaning from a healthy donor piglet that is 4 weeks older is an effective tool in altering the microbiota in piglets. Whether this change is transient or stable cannot be determined within the current study design; however, the aim of the study was to identify whether FMT could cause a change that would positively impact the pig within the period where they are most at risk to enteric dysbiosis post weaning. Hence, further studies are required to elucidate the durability of the effect of FMT beyond 14 days and to determine whether FMT has any long-lasting production outcomes. To our knowledge, this is the first study to document a change in the microbiota of piglets after a single FMT post weaning. The findings from this study provide valuable information for the development of future work investigating FMT in the post weaning period.

## Figures and Tables

**Table 1:** Genus contributing to the top 60% of significant dissimilarity of bacteria between FMT and CON at 24 days of age as determined by similarity percentages (SIMPER). Overall average dissimilarity between treatments is 27%.

					Group FMT	Group CON	
Phyla	Class	Order	Family	Genus	Average Abundance	Average Abundance	%
Actinobacteria	Coriobacteriia	Coriobacteriales	*Coriobacteriaceae*	*Collinsella*	0.26	0.27	0.69
Bacteroidetes	Bacteroidia	Bacteroidales	*Bacteroidaceae*	*Bacteroides*	0.82	0.68	1.24
			*Odoribacteraceae*	*Butyricimonas*	0.27	0.25	0.75
			*p-2534-18B5*		0.46	0.29	1.09
			*Paraprevotellaceae*	*YRC22*	0.61	0.38	0.94
				*CF231*	0.9	0.9	0.81
			*Porphyromonadaceae*	*Parabacteroides*	1.02	0.79	1.25
				*Paludibacter*	0.37	0.24	1.18
			*Prevotellaceae*	*Prevotella*	2.12	2.31	1.13
				*Prevotella*	1.19	1.03	1.09
			*Prevotellaceae*		0.69	0.58	0.82
			*RF16*		0.47	0.36	0.81
			*Rikenellaceae*		0.25	0.2	0.73
			*S24-7*		1.77	1.64	0.99
Deferribacteres	Deferribacteres	Deferribacterales	*Deferribacteraceae*	*Mucispirillum*	0.4	0.21	0.8
Elusimicrobia	Elusimicrobia	Elusimicrobiales	*Elusimicrobiaceae*		0.26	0.12	0.77
Fibrobacteres	Fibrobacteria	Fibrobacterales	*Fibrobacteraceae*	*Fibrobacter*	0.36	0.22	0.7
Firmicutes	Bacilli	Lactobacillales	*Lactobacillaceae*	*Lactobacillus*	0.46	0.5	0.98
			*Streptococcaceae*	*Streptococcus*	0.41	0.32	0.78
	Clostridia	Clostridiales	*Christensenellaceae*		1.1	0.9	1.27
			*Clostridiaceae*	*Sarcina*	0.82	0.67	1.02
			*Clostridiaceae*		0.49	0.41	0.81
				*SMB53*	0.48	0.37	0.72
			*Lachnospiraceae*		1.43	1.2	1.16
				*Shuttleworthia*	0.37	0.13	1.02
				*Dorea*	0.75	0.63	0.96
				*Butyrivibrio*	0.54	0.4	0.94
				*Lachnobacterium*	0.38	0.25	0.73
				*Roseburia*	0.84	0.93	0.69
				*Lachnospira*	0.71	0.65	0.69
			*Mogibacteriaceae*		0.82	0.72	0.8
			*Peptococcaceae*	*rc4-4*	0.33	0.26	0.66
			*Ruminococcaceae*	*Faecalibacterium*	1.01	1.14	0.91
				*Oscillospira*	1.52	1.45	0.73
			*Ruminococcaceae*		1.69	1.56	0.66
			*Veillonellaceae*	*Mitsuokella*	0.43	0.53	1.07
				*Dialister*	0.54	0.2	1.06
			*Veillonellaceae*		0.63	0.61	1
				*Acidaminococcus*	0.57	0.43	0.98
				*Megasphaera*	0.63	0.64	0.9
				*Anaerovibrio*	0.95	0.91	0.75
		Clostridiales			1.07	0.91	1.01
		Clostridiales			1.17	1.02	0.66
Firmicutes	Erysipelotrichi	Erysipelotrichales	*Erysipelotrichaceae*	*p-75-a5*	0.77	0.67	0.82
				*Catenibacterium*	0.65	0.71	0.8
Proteobacteria	Alphaproteobacteria	RF32			0.33	0.21	0.82
	Alphaproteobacteria				0.3	0.16	0.76
	Deltaproteobacteria	GMD14H09			0.72	0.68	1.1
	Epsilonproteobacteria	Campylobacterales	*Campylobacteraceae*	*Campylobacter*	0.91	0.75	1.08
	Gammaproteobacteria	Aeromonadales	*Succinivibrionaceae*	*Succinivibrio*	1.18	1.44	1.85
			*Succinivibrionaceae*	*Ruminobacter*	0.63	0.39	1.51
			*Succinivibrionaceae*		0.52	0.15	1.5
		Enterobacteriales	*Enterobacteriaceae*	*Escherichia*	0.76	0.54	1.33
		Pasteurellales	*Pasteurellaceae*	*Actinobacillus*	0.37	0.3	0.76
Spirochaetes	Spirochaetes	Spirochaetales	*Spirochaetaceae*	*Treponema*	1.31	1.16	1.14
Synergistetes	Synergistia	Synergistales	*Dethiosulfovibrionaceae*	*Pyramidobacter*	0.36	0.26	0.87
			*Dethiosulfovibrionaceae*		0.33	0.27	0.66
Tenericutes	RF3	ML615J-28			0.38	0.27	0.75
TM7	TM7-3	CW040	*F16*		0.33	0.17	0.8
Verrucomicrobia	Verruco-5	WCHB1-41	*RFP12*		0.33	0.18	0.66
WPS-2					0.31	0.21	0.74

**Table 2:** Genus contributing to the top 60% of significant dissimilarity of bacteria between FMT and CON at 35 days of age as determined by SIMPER. Overall average dissimilarity between treatments is 22%.

					Group FMT	Group CON	
Phyla	Class	Order	Family	Genus	Average Abundance	Average Abundance	%
Actinobacteria	Coriobacteriia	Coriobacteriales	*Coriobacteriaceae*	*Collinsella*	0.37	0.28	0.77
Bacteroidetes	Bacteroidia	Bacteroidales	*Paraprevotellaceae*	*YRC22*	0.21	0.28	0.9
				*CF231*	0.75	0.88	0.74
					0.7	0.55	0.69
			*Porphyromonadaceae*	*Parabacteroides*	0.52	0.59	0.94
			*Prevotellaceae*	*Prevotella*	1.19	1.3	1.12
			*S24-7*		1.32	1.3	0.82
		Bacteroidales			0.43	0.41	0.9
Deferribacteres	Deferribacteres	Deferribacterales	*Deferribacteraceae*	*Mucispirillum*	0.35	0.26	0.99
Elusimicrobia	Elusimicrobia	Elusimicrobiales	*Elusimicrobiaceae*		0.21	0.07	0.79
Fibrobacteres	Fibrobacteria	Fibrobacterales	*Fibrobacteraceae*	*Fibrobacter*	0.31	0.2	0.78
			*Streptococcaceae*	*Streptococcus*	0.45	0.32	1.01
Firmicutes	Clostridia	Clostridiales	*Clostridiaceae*	*Sarcina*	0.53	0.73	1.31
				*SMB53*	0.86	0.88	0.84
			*Clostridiaceae*		0.94	0.95	0.82
			*Lachnospiraceae*	*Shuttleworthia*	0.53	0.41	1.64
				*Lachnobacterium*	0.56	0.79	1.63
			*Lachnospiraceae*		1.32	1.4	1.39
				*Roseburia*	1.47	1.55	1.1
				*Anaerostipes*	0.4	0.32	0.87
				*Lachnospira*	0.95	1.08	0.81
				*Ruminococcus*	1.06	1.03	0.79
				*Dorea*	0.68	0.71	0.78
				*Coprococcus*	0.94	0.96	0.72
			*Peptococcaceae*	*rc4-4*	0.11	0.28	0.87
			*Ruminococcaceae*	*Faecalibacterium*	1.18	1.08	0.82
				*Oscillospira*	1.28	1.23	0.77
			*Veillonellaceae*	*Dialister*	0.91	0.34	2.29
			*Veillonellaceae*		0.71	0.66	1.43
				*Acidaminococcus*	0.53	0.34	1.4
				*Mitsuokella*	0.65	0.53	1.35
				*Megasphaera*	0.7	0.53	1.28
				*Phascolarctobacterium*	0.72	0.96	0.99
	Clostridia				0.24	0.04	0.86
	Erysipelotrichi	Erysipelotrichales	*Erysipelotrichaceae*	*Catenibacterium*	0.75	0.58	1.13
				*Bulleidia*	0.98	0.91	1.09
				*p-75-a5*	0.64	0.68	0.77
				*L7A_E11*	0.33	0.25	0.74
Proteobacteria	Alphaproteobacteria	RF32			0.36	0.45	1.09
	Betaproteobacteria	Burkholderiales	*Oxalobacteraceae*	*Oxalobacter*	0.14	0.15	0.73
		Tremblayales			0.32	0.26	1.44
	Deltaproteobacteria	GMD14H09			0.52	0.45	1.36
	Epsilonproteobacteria	Campylobacterales	*Campylobacteraceae*	*Campylobacter*	0.77	0.67	1.34
	Gammaproteobacteria	Aeromonadales	*Succinivibrionaceae*	*Succinivibrio*	0.93	0.98	1.84
			*Succinivibrionaceae*		0.47	0.2	1.7
		Enterobacteriales	*Enterobacteriaceae*	*Escherichia*	0.34	0.27	0.81
		Pasteurellales	*Pasteurellaceae*	*Actinobacillus*	0.32	0.15	0.93
Spirochaetes	Spirochaetes	Spirochaetales	*Spirochaetaceae*	*Treponema*	0.91	1.01	1.65
Synergistetes	Synergistia	Synergistales	*Dethiosulfovibrionaceae*		0.22	0.27	0.7
TM7	TM7-3	CW040	*F16*		0.38	0.41	0.93
Unclassified Bacteria					0.26	0.14	0.81
